# Validation of the diagnosis of eosinophilic esophagitis based on histopathology reports in Sweden

**DOI:** 10.48101/ujms.v126.7687

**Published:** 2021-08-13

**Authors:** Lovisa Röjler, Ida Glimberg, Marjorie M. Walker, John J. Garber, Jonas F. Ludvigsson

**Affiliations:** a Department of Pediatrics, Örebro University Hospital, Örebro, Sweden; b Department Anatomical Pathology, Faculty of Health and Medicine, School of Medicine and Public Health, University of Newcastle, Callaghan, NSW, Australia; c Gastrointestinal Unit, Massachusetts General Hospital, Harvard Medical School, Boston, MA, USA; d Department of Medical Epidemiology and Biostatistics, Karolinska Institutet, Stockholm, Sweden; e Division of Epidemiology and Public Health, School of Medicine, University of Nottingham, City Hospital, Nottingham, UK; f Celiac Disease Center, Department of Medicine, Columbia University College of Physicians and Surgeons, New York, USA

**Keywords:** Eosinophilic esophagitis, inflammation, validation, histopathology

## Abstract

**Background:**

Eosinophilic esophagitis (EoE) is a relatively new diagnosis, where until recently a specific international classification of disease code was missing. One way to identify patients with EoE is to use histopathology codes. We validated the clinicopathological EoE diagnosis based on histopathology reports and patient charts to establish these data sources as the basis for a nationwide EoE patient cohort.

**Methods:**

Through the Epidemiology Strengthened by histoPathology Reports in Sweden (ESPRESSO) study, we randomly selected 165 patients from five Swedish health care regions with a histopathologic diagnosis of EoE. Patients were assigned a histopathology diagnosis of EoE if they had ≥15 eosinophils per high-power field or, in the absence of eosinophil quantification, the pathologist interpreted the biopsy as consistent with EoE. Patient charts were scrutinized to see if the other diagnostic criteria were fulfilled. Of the 131 received patient charts, 111 (85%) had sufficient information to be included in the study.

**Results:**

Of the 111 validated patients, 99 had EoE, corresponding to a positive predictive value of 89% (95% confidence interval = 82–94%). Dysphagia was the most common symptom (*n* = 78, 70%), followed by food impaction (*n* = 64, 58%) and feeding difficulties (*n* = 37, 33%). Twelve patients had coexisting asthma (11%) and 16 allergic rhinitis (14%). Seventeen patients underwent esophageal dilatation (15%), of which seven had more than one dilatation. Ninety-seven (87%) patients had a proton-pump inhibitor treatment ≤2 years before or after the diagnosis. Forty-two patients (38%) had been prescribed inhalation steroids and 64 (58%) had undergone esophageal radiology.

**Conclusion:**

Histopathology reports from the ESPRESSO cohort with esophageal eosinophilic inflammation are suggestive of EoE.

## Introduction

Eosinophilic esophagitis (EoE) has been recognized relatively recently and was first proposed as a distinct clinicopathologic entity in 1993–1994 (1, 2). Time trends in EoE incidence and prevalence have shown an exponential rise in the past 25 years (3). EoE is a chronic, local immune-mediated esophageal disease of the squamous esophagus. It is clinically characterized by symptoms related to esophageal dysfunction, endoscopic findings of rings, linear furrows, exudates, edema, strictures, narrowing and crepe-paper mucosa on biopsy ≥15 eosinophils per high-power field (HPF, 60 eosinophils/mm^2^), and eosinophilia isolated to the esophagus (4). Endoscopy can also be macroscopically normal (5).

The diagnosis of EoE requires that clinical manifestations and pathologic data be interpreted in tandem (6). The diagnosis of EoE has traditionally been limited to proton-pump inhibitor (PPI) non-responders, but guidelines from an international consensus meeting in 2017 acknowledged that PPI therapy is an appropriate and effective treatment for a significant proportion of EoE patients. Thus, PPI non-response as a diagnostic criterion for EoE has since been removed (6). EoE and gastroesophageal reflux disease (GERD) may coexist with significant overlap in symptoms, and therefore, it can sometimes be difficult to distinguish between EoE and GERD on clinical grounds alone (7, 8).

In register-based research, patients of interest are usually identified through a relevant international classification of disease (ICD) code. However, in the case of EoE, no specific ICD-code was available until 2012 in Sweden, and this prohibits studies of long-term prognosis in EoE. We envisioned using an alternative means to identify EoE patients: histopathology reports with a Systematized Nomenclature of Medicine-Clinical Terms (SNOMED-CT) code T62 (esophagus) in combination with M47150 (eosinophilic inflammation) from the Epidemiology Strengthened by histoPathology Reports in Sweden (ESPRESSO) cohort study (9). In the current paper, we aimed not only to validate esophageal eosinophilia against EoE diagnosis in a randomly selected group of patients but also to characterize these patients with regards to symptoms, investigations, histopathology, laboratory data, differential diagnoses, smoking and alcohol consumption, and treatment.

## Materials and methods

### Study population

Swedish biopsy data are categorized according to the SNOMED-CT system, a system of comprehensive health and clinical terminology used in many countries. In a nationwide project, we collected gastrointestinal histopathology report data from all pathology departments in Sweden (*n* = 28) from the time period 1965 to 2017; this cohort forms the ESPRESSO study (9) and contains more than 6 million biopsy reports.

Through searching ESPRESSO for individuals with a biopsy from the esophagus (T62) that showed inflammation with eosinophil infiltration (M47150) (*n* = 1,663), we aimed to establish a cohort of patients with EoE. From this cohort, an external biostatistician randomly selected 165 patients from 17 hospitals in five health care regions in Sweden that included both local and university hospitals: Örebro (departments of medicine, surgery, and ear-nose-throat (ENT) and private clinic Läkargruppen), Karlskoga (medicine and surgery), Lindesberg (surgery), Norrtälje (surgery and endoscopy), Karlstad (surgery), Torsby (surgery), Skövde (ENT and surgery), Stockholm Karolinska (ENT, gastroenterology, and endoscopy), Stockholm Södersjukhuset (medicine), Eksjö (endocrinology and gastroenterology), Arvika (surgery), Danderyd (emergency department), Lidköping (medicine), Falun (medicine), and Jönköping (surgery).

The departments responsible for each patient with a T62 topography code and an M47150 morphology code (mainly internal medicine, surgery, or ENT) were contacted, and patient charts were requested, which included discharge notes, histopathology reports, laboratory data, endoscopy notes, radiology reports, and surgery notes. Between October 2017 and August 2018, we received clinical data from 131 individuals (79%). The patient charts of these individuals were then reviewed using a standardized form based on similar validation studies of inflammatory bowel disease (IBD) (10) and microscopic colitis (11), but adjusted to the unique conditions of EoE. Additional symptoms, laboratory data, radiology, endoscopies, concomitant diseases (such as allergy), gastrointestinal infections, and ongoing treatment (medical or diet) were examined (see Supplementary material for list of variables). The patients had sought health care for their medical complaints between December 2000 and January 2017, and the charts originated from patients’ hospital visit appointments between 1989 and 2017.

Biopsies from the esophagus were categorized as ‘upper esophagus’ or ‘lower esophagus’. Biopsies taken from the mid-esophagus as well as those taken 0–35 cm from the teeth were considered as ‘upper esophagus’, and biopsies taken from >35 cm were considered as ‘lower esophagus’. When data on location were inexact, biopsies were classified as from the ‘upper esophagus’.

Relevant data (a symptom, an examination, or other information) were considered absent if not explicitly reported in the patient charts. For instance, a patient without a record of dysphagia in the patient chart was interpreted as having ‘no dysphagia’.

### Case definition

**Figure 1:**
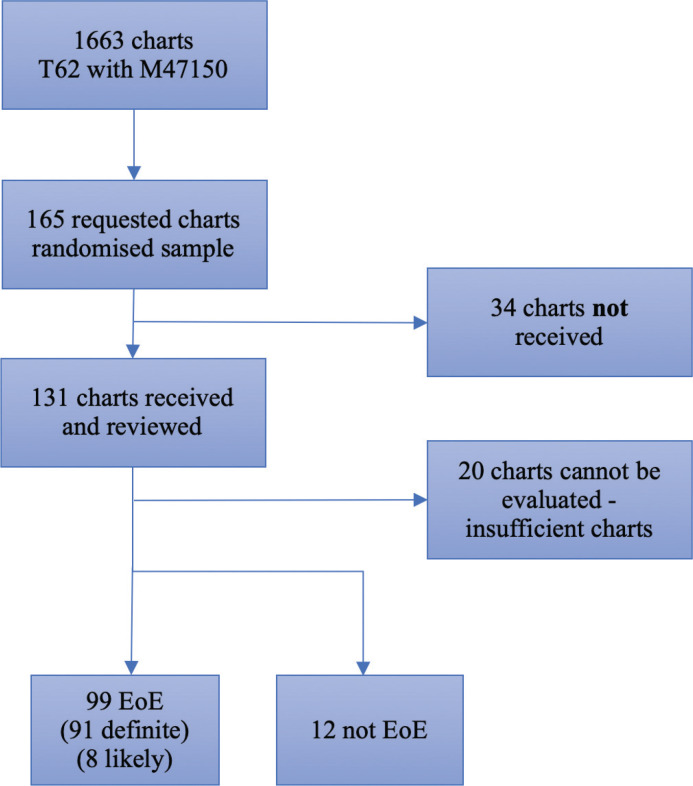
Study Flowchart of patients with a histopathology diagnosis of eosinophilic esophagitis (EoE).

LP and IG classified cases as *definite, likely* EoE (these two categories were merged for the positive predictive value [PPV] calculations) or *negative* for EoE. *Likely* was defined as borderline number of eosinophils, but where other supporting features such as typical endoscopic appearance or highly suspicious clinical symptoms were present. In case of uncertainty, JJG was consulted. Patients with insufficient patient chart data were excluded from the study (Figure 1).

### Ethical approval

This study was approved by Ethical Review Board in Stockholm (July 19, 2017; reference number: 2017/1497-32). In accordance with the ethics approval, no patient was contacted in person since this study was part of a larger register-based project and aimed to verify already collected histopathology data (12).

### Statistics

This is a cross-sectional descriptive study. The random selection of patients was performed using R (version 3.6.1, R Foundation for Statistical Computing, Vienna, Austria). PPVs and 95% confidence intervals (CIs) were calculated using EpiTools (https://epitools.ausvet.com.au/ciproportion?page=CIProportion, accessed October 2020) and the Wilson score interval.

When *P*-values were calculated for comparison between symptoms in true and false-positive EoE individuals, Pearson Chi-square and Fishers exact test were used. Our alpha level (significance level) was 0.05.

### Sample size determination

To detect a PPV of 90% with a 95% CI of 85–95%, we needed 139 patients (epitools.ausvet.com.au). From experience, we know that not all charts can be found or are delivered, and we, hence, requested 165 patient charts from the clinics.

## Results

We received patient charts from 131 individuals with a histopathology code for eosinophilia in the esophagus (M47150). In 111 (85%), there were sufficient data to confirm or reject the diagnosis of EoE.

Of the 111 patients, 91 (82%) had definite EoE and eight (7%) likely EoE. Twelve patients (11%) were defined as ‘not EoE’ based on the lack of an adequate number of eosinophils (<15/HPF) (*n* = 9), another primary esophageal disorder such as Barrett’s esophagus (*n* = 2), and dysphagia primarily attributable to poor dentition (*n* = 1). Lack of clinical notes was the only reason for excluding 20 patients from the study. The outcome of the chart review is illustrated in Figure 1. Hence, of the 111 patients with available data, 99 had EoE, corresponding to a PPV of 89% (95% CI = 82–94%). Two out of 111 patients had a family history of EoE.

### Clinical characteristics and presenting symptoms

The median age at first diagnostic biopsy was 63 years (range 12–87 years; one child). Of the 111 patients included in the study, the majority were males (*n* = 86 (78%)). Duration of symptoms was listed in 79 patient charts, and 23% (*n* = 25) of the patients had had symptoms for >10 years. Smoking (current or past) was noted in 15 patients (14%) and alcohol use in 24 (22%) (Table 2).

Body mass index (BMI) was recorded in nine patients (range 20–34.2 kg/m^2^). Twelve patients had coexisting asthma (11%) and 16 allergic rhinitis (14%). Atopic dermatitis was noted in five patients (5%). Thirteen patients (12%) had a record of food allergies.

Dysphagia was the most common symptom (*n* = 78, 70%), followed by food impaction (*n* = 64, 58%) and feeding difficulties (*n* = 37, 33%) (Table 1).

**Table 1:** Patient symptoms reported in charts among 111 patients with a histopathology code consistent with eosinophilic esophagitis (EoE).

Symptom	All patients (*n* = 111)	Patients with true EoE (*n* = 99)	Patients with no EoE (*n* = 12)
Dysphagia, *n* (%)	78 (70)	71 (72)	7 (58)
Food impaction, *n* (%)	64 (58)	61 (62)	3 (25)
Feeding difficulties, *n* (%)	37 (33)	33 (33)	4 (33)
Vomiting, *n* (%)	19 (17)	19 (19)	0 (0)
Abdominal pain, *n* (%)	17 (15)	15 (15)	2 (17)
Weight loss, *n* (%)	12 (11)	10 (10)	2 (17)
Other pain, (%)			
Throat	2 (2)	2 (2)	0 (0)
Sternal	12 (11)	12 (12)	0 (0)
Epigastric	2 (2)	1 (1)	1 (8)
Abdominal	4 (4)	4 (4)	0 (0)
Headache/backpain	2 (2)	1 (1)	1 (8)
Myoclonus unspecified location	1 (1)	0 (0)	0 (0)
Eating slowly	6 (5)	6 (6)	0 (0)

Comparing patients with true EoE versus those with false-positive EoE (not confirmed through patient chart review) revealed that food impaction (*P* = 0.015) was more common in true-positive EoE (Supplementary Table S1).

### Radiology and endoscopic examinations

Of the 111 EoE patients, 64 (58%) underwent not only esophageal radiology (true EoE vs. not EoE: *n* = 56 vs. 8), mainly barium esophagram (*n* = 41; 36 vs. 5) but also computed tomography (*n* = 5; 4 vs. 1) and conventional X-ray (*n* = 3; 3 vs. 0). Fifteen patients (14%; 13 vs. 2) underwent unspecified diagnostic radiology.

Many of the examinations were performed in the setting of acute food impaction (*n* = 17), and six examinations demonstrated strictures of the esophagus. One patient had a perforation after dilatation, and one had esophageal achalasia. Twenty-five patients (23%) underwent esophageal manometry and pH registration. In the manometry examinations, 13 were normal, two showed suspected achalasia (these two patients fulfilled our EoE criteria), five hypomotility, two hypertensive peristalsis, one insufficient lower esophageal sphincter, and two were inconclusive. pH registration indicated normal pH in eight patients, reflux in three (pH < 4 for a significant part of the day), mild reflux in three, and 11 were without result.

All patients underwent endoscopy with biopsy at least once. In 54 patients (49%), the location and number of biopsies could be determined from the endoscopy reports. Among these, the majority (54%) had biopsies taken from both the upper and lower esophagus, although some patients had biopsies taken from only the upper (20%) or lower (26%) esophagus. When this could be determined from the endoscopy reports, the mean number of biopsies taken was 2 (range 1–9) from the upper esophagus and 2 (range 1–8) from the lower esophagus.

Five patients (5%) had a record of *Helicobacter pylori* positivity. No patient was positive for Giardia. A detailed laboratory data can be found in the Supplementary material.

### Treatment

Treatment data are presented in Table 2. Thirteen patients (12%) were on PPI therapy at the time of diagnostic biopsy, and 97 patients (87%) had a PPI trial ≤2 years before or after the diagnosis. Dietary advice had been recorded in five patients, but none of these were recommended an empiric elimination diet (e.g. six-food elimination diet, SFED) or elemental diet. Dietary advice instead included avoidance of confirmed allergens or foods with specific textures (e.g. large pieces of meat). One patient was already avoiding meat products due to dysphagia.

**Table 2:** Drugs prescribed to eosinophilic esophagitis (EoE) patients within 2 years before or after the first biopsy of EoE.

Medication	*N* = 111 (%)
PPI treatment, *n* (%)	97 (87)
PPI at the time of first biopsy, *n* (%)	13 (12)
Response to PPI treatment, *n* (%)	50 (45)
Other antacids, *n* (%)	
Ranitidine	6 (5)
Aluminum-hydroxide antacid	12 (11)
Oral steroids, *n* (%)	
Betamethasone	4 (4)
Prednisolone	4 (4)
Swallowed steroids, *n* (%)	
Mometasone	27 (24)
Fluticasone/flixotide	11 (10)
Budesonide	4 (4)
Antihistamines, *n* (%)	
Desloratadine	6 (5)
Loratadine	2 (2)
Others:	
Montelukast, *n* (%)	6 (5)
Omalizumab, *n* (%)	1 (1)
Azathioprine, *n* (%)	0 (0)
Other biologics, *n* (%)	0 (0)
Other drugs, *n* (%) (list restricted to drugs prescribed to at least three patients)	Paracetamol, simvastatin, amlodipine, cocillana-etylmorphine, acetylsalicylic acid, betamethasone, enalapril, zopiclone, phenoxymethylpenicillin, metformin, metoprolol, propiomazine, atorvastatin, ipratropium, ciprofloxacin, paracetamol/codeine, levothyroxine, insulin, oxycodone, ramipril, pivamdinocillin

PPI: proton-pump inhibitor.

The most common EoE treatments were swallowed steroids and esophageal dilation. Seven patients (6%) had received systemic steroids (betamethasone or prednisolone). Swallowed inhalation steroids were prescribed to 42 patients (38%). The most common swallowed steroids were mometasone furoate (nasonex, *n* = 27), fluticasone propionate (*n* = 11), and budesonide (*n* = 4). Three patients (3%) had budesonide/formoterol prescribed but for asthma indication. One patient had intraesophageal steroid injection at the time of dilatation. In addition, six patients (5%) were prescribed montelukast. No patient had a record of biological treatment.

Seventeen patients (15%) underwent therapeutic esophageal dilatation; seven (6%) of these had undergone ≥2 dilatations.

In the year before biopsy, 13 (12%) patients had been prescribed antibiotics for indications of respiratory tract infection, skin wound, prophylaxis for bladder tumor resection, otitis media, erysipelas, perforated esophagus, and unknown reasons (*n* = 7). Twenty patients (18%) had a record of non-steroidal anti-inflammatory drug use ≤1 year before or after the diagnosis of EoE.

## Discussion

A major criterion for the diagnosis of EoE is the presence of at least 15 eosinophils/HPF in the esophageal mucosa, regardless of the results of PPI treatment outcome (4, 13). Eosinophils are found throughout the gastrointestinal mucosa but are typically not present in the normal esophagus (14). Based on this information, we hypothesized that the presence of eosinophils in adequate numbers to trigger a histopathologic diagnosis of EoE would, in the absence of other plausible explanations for esophageal eosinophilia, be highly predictive of a valid clinical diagnosis of EoE. In the current study, we examined a random subset of 111 patients with a histopathology report with eosinophilia in the esophagus and found that 99 (89%) had a clinicopathological diagnosis of EoE – meeting all three criteria for EoE. This validity is similar to having a physician-assigned diagnosis in the Swedish Patient Register (15).

### Main findings and comparison with earlier literature

In accordance with earlier research (3, 4), most patients (78%) in our nationwide cohort study were male. The majority had typical symptoms and presentation of EoE, including chronic dysphagia (70%) and a history of prior food impaction (58%), with very few patients reporting prominent heartburn. Taken together, these results suggest a patient population quite distinct from that afflicted with chronic GERD although these two medical conditions often overlap. With these symptoms, it is likely that patients suffer from a reduced health-related quality of life (16). Moreover, it is probable that the prevalence and severity of esophageal symptoms reported in our study are underestimated because lack of any record for a specific symptom in the patient chart was interpreted as missing. Slow eating and excessive chewing may represent coping strategies that EoE patients consciously or subconsciously employ to avoid food impaction and ease dysphagia (17). Extended mealtime (slow eating) was only reported in six patients (5%) with EoE in our study. This symptom may be more common but overshadowed by dysphagia and food impaction, which patients may perceive as more serious concerns. We also cannot rule out that Swedish physicians do not ask questions specifically about the amount of time taken to eat a meal, especially when they have confirmed the presence of dysphagia.

The average age at first biopsy in our study was 63 years, which is higher than expected. EoE has been described in all ages, but most studies examining EoE in adults have reported an average age of diagnosis of 30–50 years (18–23).

There are several possible explanations for the more advanced age observed in our study. First, we documented only the date of the index esophageal biopsy in which increased eosinophils were noted leading to the histopathological diagnosis, and not the date from which symptoms first became apparent, although we found that 53% of patients had experienced symptoms for >2 years and 23% for >10 years.

For cases with adequate follow-up assessment, just under half (45%) were documented to achieve a good clinical response. This finding agrees with published data describing rates of PPI-induced remission in 30–50% (24). Also in line with previous studies (25), 38% of EoE patients in our study were treated with swallowed steroids (mometasone, fluticasone, and budesonide). The high rate of mometasone use in our study population may reflect that mometasone is listed, along with fluticasone, as a topical steroid option with more local effect and less systemic exposure compared with budesonide in Swedish EoE clinical practice guidelines (26).

There may be several reasons for the low rate of dietary therapies. First, it may reflect that the majority of patients in our study were primarily treated by a gastroenterologist or internist (rather than an allergist), specialties that may have a lower comfort level with recommending dietary therapy. Second, patients opting for dietary therapy will undergo numerous repeat endoscopies during the process of empiric elimination and step-wise food reintroduction. For example, it is estimated that patients choosing an empiric SFED will undergo a mean of seven upper endoscopies with a predicted success rate of 55–60% in ultimately defining their dietary trigger(s) (27). The frequency of therapeutic elimination diets in our study may also be underestimated given that dieticians have not traditionally recorded interventions in physician patient charts.

Only a minority of patients had a record of allergic comorbidity. Twelve patients (11%) had asthma, which was lower than expected considering the strong allergic component in EoE (28). In another study conducted in the US (29), 24% of adult EoE patients and 52% of pediatric EoE patients were diagnosed with asthma, figures considerably higher than in our study. The most likely reason for this discrepancy is that physicians in routine health care (as opposed to those in a research setting) may not register the presence of comorbidity, or that such diseases are cared for outside the clinic that managed the EoE diagnosis.

The major strengths of our study include its population-based design and real-world setting. However, this paper also has a number of limitations. First, we used routine care data, which means that some information for comorbidities, medication, BMI, smoking, and alcohol consumption may not have been documented by the treating physician (as opposed to a research setting). A second limitation is our attrition rate. Of the 131 patient charts, only 111 had sufficient data to be included in the analyses. None of the hospitals included in this study was situated in the North of Sweden, and, hence, our study has limited information on geographical differences of EoE within Sweden. Third, we acknowledge that an EoE ICD code was introduced in the Swedish Patient Register in 2012. Much of our data, however, originate from before 2012; taking advantage of biopsy reports to identify EoE in the early 2000s allows researcher to carry out cohort studies of long-term prognosis of EoE, which is not feasible if limited to patients diagnosed in 2012 or later. Fourth, in a subset of patients without exact data on the number of eosinophils/HPF, an EoE diagnosis was sometimes accepted when the pathologist had interpreted that the biopsy was consistent with EoE. This is not according to current practice (30), but was done since a large proportion of esophageal biopsies were carried out in the early 2000s when current diagnostic criteria were not yet established.

Fifth, we lacked data on the location of a subset of esophageal biopsies, and we urge caution when interpreting findings related to upper as opposed to lower esophagus. Sixth, because of restrictions imposed by the Ethics Review Board, we were unable to rereview the slides of the patients examined for suspected EoE. Still, other studies have shown a 90% accuracy when reanalyzing biopsies for number of eosinophils per HPF (31). Seventh, ideally, data on histopathological features other than number of eosinophils per HPF should have been reported in this review (such as basal zone hyperplasia, eosinophil abscesses, eosinophil surface layering, dilated intracellular spaces, surface epithelial alteration, dyskeratotic epithelial cells, and lamina propria fibrosis) (32). Unfortunately, such information was rarely available in the biopsy reports (which tended to focus on eosinophil numbers) and, hence, not recorded in this study. Finally, in many EoE patients, there was a lack of detailed data on endoscopic appearance.

## Conclusion

Histopathology reports from ESPRESSO cohort indicating eosinophilic inflammation in the esophagus are suggestive of EoE.
